# Quantifying the influence of a permanent shade pavilion structure on WBGT and activity modification

**DOI:** 10.1007/s00484-026-03345-8

**Published:** 2026-10-28

**Authors:** Andrew Grundstein, Susan Yeargin, Earl Cooper, Matthew Fuller, Shea Wilson-Gregg

**Affiliations:** 1 Department of Geography, University of Georgia, Athens, 30602 GA USA; 2 Exercise Science Department, University of South Carolina, Columbia, 29208 SC USA; 3 Department of Kinesiology, University of Georgia, Athens, 30602 GA USA; 4 Athens YMCA, Athens, 30606 GA USA

**Keywords:** Heat mitigation, Shade structures, Outdoor sports, Outdoor recreation, Wet bulb globe temperature (WBGT)

## Abstract

Exposure to solar radiation significantly elevates heat stress during outdoor physical activity, increasing the risk of exertional heat illness. This study quantified the operational impact of a permanent, full-coverage shade pavilion on wet-bulb globe temperature (WBGT) and evaluated how shade-induced cooling modifies activity-restriction thresholds in a recreational sport setting. In Athens, Georgia, USA, we collected 309 paired sun–shade observations across a wide, representative range of humid subtropical summer conditions, comparing a sun-exposed grass field with the shaded pavilion area. WBGT values were classified according to Georgia High School Association (GHSA) heat-safety guidelines.

The pavilion reduced WBGT by an estimated 4.1°C relative to the sun-exposed grass site under mean observed meteorological conditions, although the magnitude of this cooling varied with sky cover, with larger reductions under sunnier skies. In full sun, only 36% of observations fell into the no modification category, and 15% reached the most restrictive thresholds. Under the pavilion, 96% of observations required no modification, and none reached the highest activity restriction categories. These shifts in activity-modification categories translate into substantially longer durations of unrestricted activity under the pavilion, corresponding to approximately 6.7 versus 2.5 h during a standardized 9 a.m. to 4 p.m. period in shaded and sun-exposed areas, respectively.

These findings demonstrate that permanent shade infrastructure can serve as an effective heat mitigation tool for schools, parks, and municipalities. The exact reductions in WBGT and associated changes in activity modification decisions, however, may vary depending on the specific shade structure design and regional climate setting.

**Supplementary Information:**

The online version contains supplementary material available at https://doi.org/10.1007/s00484-026-03345-8.

## Introduction

Exposure to hot weather poses a significant risk to athletes and recreational participants (Endres et al. 2019; Kucera et al. 2024). To mitigate heat hazards, sports and recreation programs are increasingly installing open-air shade structures over courts, practice fields, and multi-use recreation spaces. Examples from across the United States include shade installations at athletic facilities in Texas school districts, shaded playgrounds in large urban districts such as the Los Angeles Unified School District, and shaded public courts in municipalities such as Reno, Nevada (Dallas Innovates 2023; KFDM 2025; SpectrumNews1 2025; City of Reno 2025). These efforts reflect a broader trend of integrating shade infrastructure into heat mitigation and climate adaptation strategies.

Shading is well-established as an effective way to reduce heat stress, with research demonstrating benefits across a variety of shade types, including trees, shade sails, umbrellas, and urban structures (e.g., Vanos et al. 2017; Watanabe and Ishii 2017; Lee et al. 2018; Middel et al. 2021; Zhang et al. 2023; Aleksandrowicz and Pearlmutter 2023; Lam et al. 2023; Ouyang et al. 2024). The impact on heat stress, however, can vary depending on the type of shade structure, with greatest reductions during the day from urban structures (e.g., tunnels, breezeways, overhangs), followed by trees and lightweight structures (e.g. umbrellas, shade sails) (Lee et al. 2018; Middel et al. 2021). Because built structures generally provide larger cooling effects than trees or lightweight shading devices, permanent shade pavilions may be particularly promising for protecting outdoor participants from heat-related hazards. Despite the growing adoption of shade structures across athletic and recreational facilities in the United States, little is known about how permanent pavilion-style shade structures influence wet-bulb globe temperature (WBGT) and heat safety-decisions under real-world sports and recreation conditions. In sports settings, WBGT is widely used to guide operational decisions, including modifying practice intensity and duration, scheduling hydration breaks, and determining when activities should be restricted or cancelled (Casa et al. 2015; Roberts et al. 2023). Yet, few studies have examined how much permanent shade pavilions reduce WBGT, how this cooling effect varies under different sky conditions, or how often shading alters activity-modification decisions based on established heat-safety guidelines. Addressing these questions is increasingly important given the substantial costs associated with shade infrastructure (School District Five of Lexington & Richland Counties 2024; KFDM 2025) and the need to determine whether these investments deliver meaningful heat-mitigation benefits.

This study examines the influence of a permanent shade pavilion on WBGT in an outdoor recreational setting in the southeastern United States. Using high-frequency measurements collected under shaded and unshaded conditions, it evaluates the reduction in WBGT achieved by a full-coverage pavilion in a humid subtropical climate and its implications for activity-modification decisions. By linking microclimate modification directly to operational heat-safety thresholds, the study provides evidence relevant to heat-mitigation planning and investment decisions for athletic and recreational facilities. Specifically, the objectives of this study are to: (1) quantify the reduction in WBGT achieved by a permanent pavilion-style shade structure relative to direct sun exposure; (2) evaluate how WBGT reductions vary under differing sky-cover conditions; and (3) determine how shading influences activity-modification recommendations and usable outdoor programming time based on Georgia High School Association (GHSA) heat-safety guidelines.

## Methods

**Fig. 1:**
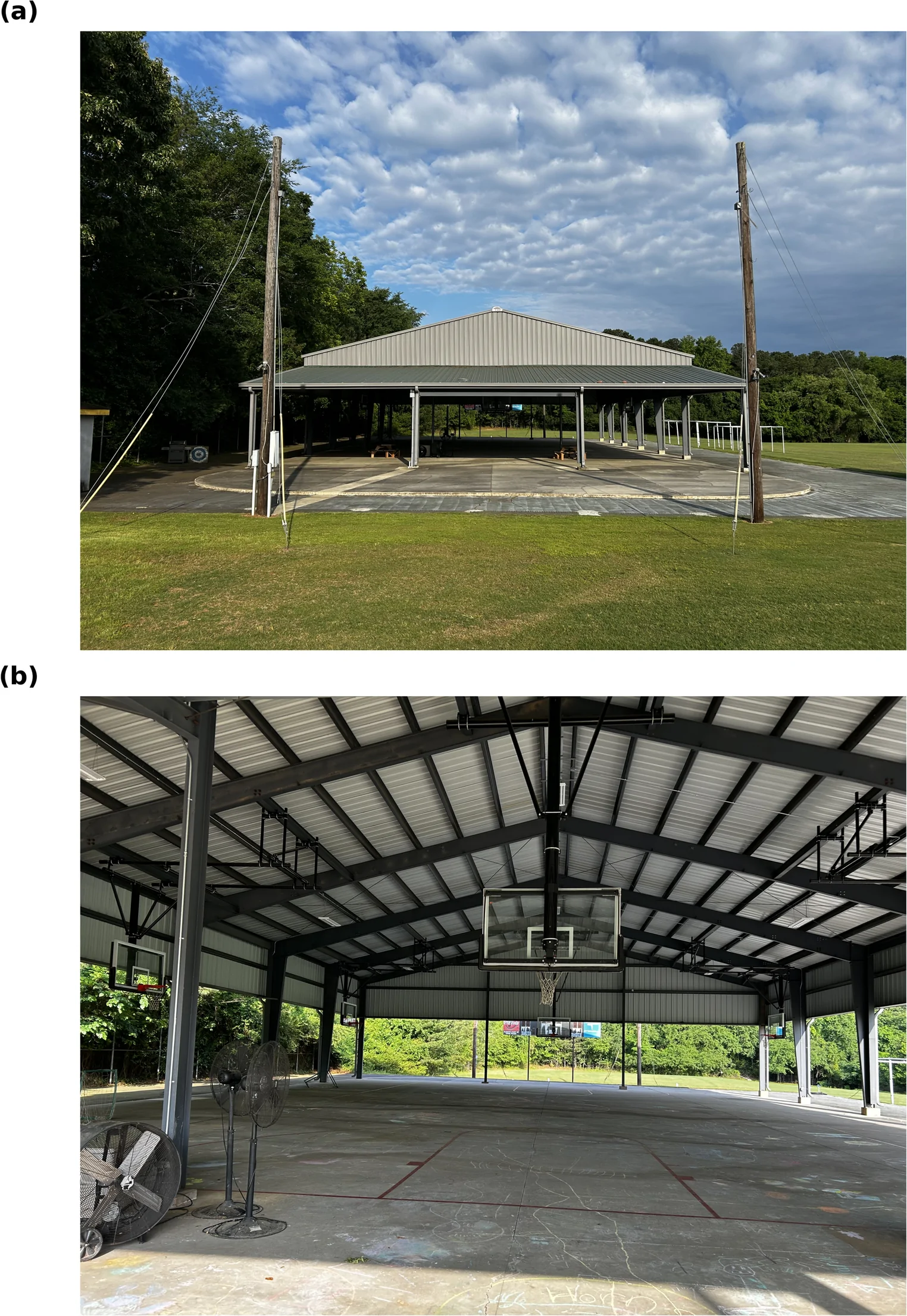
YMCA shade pavilion showing (**a**) exterior and (**b**) interior views of the shaded area

The study was conducted at a YMCA outdoor activity space in Athens, GA, U.S. Paired meteorological measurements were collected at two adjacent locations: a sun-exposed natural grass field and an adjacent 23 m x 39 m permanent shade pavilion with a concrete surface (Fig. 1). This pavilion provided continuous shade over the measurement area. The structure consists of an uninsulated metal roof on an open frame, featuring an eave height of 6.10 m and a central ridge height of 8.38 m. A 2.44 m metal siding band extends downward from the eaves, leaving a 3.66 m lower opening for ambient airflow.

Meteorological data were collected over seven days during the late spring and summer of 2025, with measurements recorded at 10-minute intervals. In total, 309 paired observations were obtained from the sun-exposed and shaded locations (Table 1). Measurements included wet bulb globe temperature (WBGT) and its component variables [i.e., black globe temperature (GT), natural wet bulb temperature (NWB), and dry bulb temperature (DB)] as well as dewpoint temperature and wind speed. WBGT was selected as the primary heat stress metric because it is widely used in sports settings to guide activity modification decisions (Casa et al. 2015; Hosokawa et al. 2019; Roberts et al. 2023). The WBGT was computed as a weighted average of the DB, NWB, and GT using the following equation:

**Table 1:** Data collection dates and meteorological conditions in the sun (average, maximum, minimum). Tair is ambient air temperature, Tdew is dewpoint temperature, and the Radiative Effect is the difference between the globe and ambient air temperatures

Date	Collection Time	Tair (^o^C)	Tdew (^o^C)	Radiative Effect (^o^C)
5/24/2025	8:40a-4:00p	23.1/29.8/17.3	12.5/15.8/8.9	9.4/15.4/4.2
6/21/2025	8:30a-4:10p	31.3/36.9/25.4	21.1/23.2/19.5	12.6/17.6/1.6
6/28/2025	8:40a-4:00p	30.9/35.8/25.5	21.2/23.2/19.5	12.9/18.3/4.5
7/12/2025	8:30a-3:00p	30.5/34.0/26.1	24.1/30.0/22.4	10.4/16.9/0.8
7/19/2025	8:40a-4:00p	29.6/34.2/26.4	23.7/25.2/22.1	12.1/18.2/6.0
8/09/2025	8:30a-4:00p	26.3/28.1/22.4	20.6/21.9/19.5	6.8/16.7/2.9
8/16/2025	8:30a-3:40p	28.9/34.0/22.7	23.8/26.9/21.2	10.2/19.2/2.1

$$\:\mathrm{WBGT}=0.7\times\:\mathrm{NWB}+0.2\times\:\mathrm{GT}+0.1\times\:\mathrm{DB}$$

All measurements were collected on Saturdays to minimize disruption to YMCA member use of the field and shade pavilion. Data were obtained using Kestrel 5400 sensors (Nielsen-Kellerman, Boothwyn, PA, USA), which were mounted on tripods at a height of 1.2 m above the surface at both locations. The shaded sensor was positioned approximately 1.5–2 m from the edge of the pavilion to avoid interference with facility use. Prior to data collection, the sensors were allowed to equilibrate for 15 min, in accordance with manufacturer recommendations (Kestrel 2025).

A radiative effect variable was calculated as the difference between globe temperature and dry bulb temperature (GT − DB), following Bernard and Iheanacho (2015), to aid interpretation of WBGT differences between shaded and sun-exposed conditions. This variable is zero if there is no radiative effect and increases as the globe temperature exceeds the dry bulb temperature. Bernard and Iheanacho (2015) describe low radiant heat effects as ≤ 3.89°C (7^o^F) and high radiant heat effects as ≥ 7.22°C (13^o^F), with peak values reaching12.22°C (22^o^F) in direct sunlight.

Environmental differences between sun and shade conditions were assessed using scatter plots and descriptive summary statistics of the measured variables. Paired differences (sun–shade) were calculated for each variable, and 95% confidence intervals around the mean difference were constructed to evaluate differences in environmental conditions (Wilks 2011). The effective sample size (nₑ) was computed from the lag-1 autocorrelation coefficient to account for temporal correlation in the data (Wilks 2011). Lag-1 autocorrelation coefficients ranged from 0.23 to 0.47, corresponding to effective sample sizes ranging from 112 to 192 observations. All confidence intervals reported in the Results are based on these effective sample size adjustments. Differences were considered statistically significant if the 95% confidence interval did not include zero (Julious 2004).

We used a linear mixed-effects model to evaluate the sun–shade WBGT difference, controlling for microclimatic variability between locations. The model took the form:

$$\begin{array}{c}WBGT_{diff}=\beta_0+\beta_1(\text{wind speed})\\+\beta_2(\text{dewpoint temperature})\\+\beta_3(\text{air temperature})+u_{day}+\varepsilon\end{array}$$

where WBGT_diff is the sun–shade WBGT difference, u_day_ is a random intercept for observation day to account for the non-independence of observations collected under the same daily meteorological conditions, and ε is the residual error term capturing remaining within-day variability. Fixed effects included wind speed, dewpoint temperature, and air temperature, selected because these variables differed measurably between the sun-exposed and shaded locations (Sect.  3.3) and are known to influence WBGT independently of radiation. Thus, the model intercept represents the adjusted mean sun–shade WBGT difference at mean observed meteorological conditions. Multicollinearity among fixed effects was assessed using variance inflation factors (VIF; O’Brien 2007). All VIF values were below 2.0 (wind speed = 1.10, dewpoint temperature = 1.77, air temperature = 1.90), suggesting that multicollinearity was unlikely to materially affect parameter estimation. The model was fit using restricted maximum likelihood (REML) estimation and statistical significance for fixed effects was evaluated at α = 0.05.

Additionally, we used a case-study approach to demonstrate how weather variability affects the magnitude of sun-shade WBGT differences. To capture both the effects of overall sky conditions (e.g., clear vs. cloudy days) and short-term variability associated by transient cloud cover, we selected the days with the largest and smallest average sun–shade WBGT differences. Examining the full diurnal WBGT time series for these contrasting days allowed us to also assess how transient cloud cover modified sun–shade differences over time. For each case-study day, we compared the WBGT at the sun and shade sites and examined the radiative component contributing to the observed differences.

Finally, we identify how observed differences in WBGT between sun-exposed and shaded sites translated into differences in recommended activity modification. Activity modification categories were determined using the Georgia High School Association (GHSA) Practice Policy for Heat and Humidity (GHSA 2025; supplemental Table S.1), a set of data-driven guidelines required for all Georgia high school sports programs (Cooper et al. 2020). Under this policy, schools measure on-site WBGT and apply the corresponding activity modification categories to planned activities. A chi-square test of independence (α = 0.05) was performed to assess whether activity modification categories differed between sun-exposed and shaded sites.

## Results

### Meteorological setting

The study area is located in a humid subtropical climate characterized by hot, humid summers. Air temperatures measured during the 8:30 a.m.– 4:00 p.m. sampling period spanned a wide variety of conditions, with morning values ranging from 17.3°C to 26.4°C and afternoon maximum temperatures ranging from 28.1°C to 36.9°C (Table 1). These conditions encompassed and exceeded the long-term (1991–2020) monthly average daytime maximum temperatures recorded at the Athens Ben Epps Airport Automated Surface Observing System (ASOS) station, located approximately 8.5 km from the study site, where monthly averages ranged from 27.9°C in May to 33.3°C in July (NCEI 2025). Study-site average daytime dewpoint temperatures during the study period ranged from 12.5°C to 24.1°C, spanning and occasionally exceeding the corresponding long-term daytime averages at the ASOS station (14.6°C in May to 20.6°C in July). Because on-site measurements of sky cover were not available, study-day cloud conditions were characterized using ASOS observations and compared with long-term monthly daytime averages. Study-day sky cover ranged from 30% to 95%, compared with long-term monthly daytime averages of 39–43%, indicating that observations captured conditions ranging from relatively clear to heavily overcast skies. This variability produced a broad range of radiative environments, with the mean radiation effect reaching 10.6°C and individual values ranging from 0.8°C to 19.2°C (Table 1). Collectively, the observations encompass a broad range of temperature, humidity, sky-cover, and radiative conditions characteristic of, and at times more extreme than, those typically experienced during the warm season in the region.

### WBGT comparison between sun-exposed and shaded

**Fig. 2:**
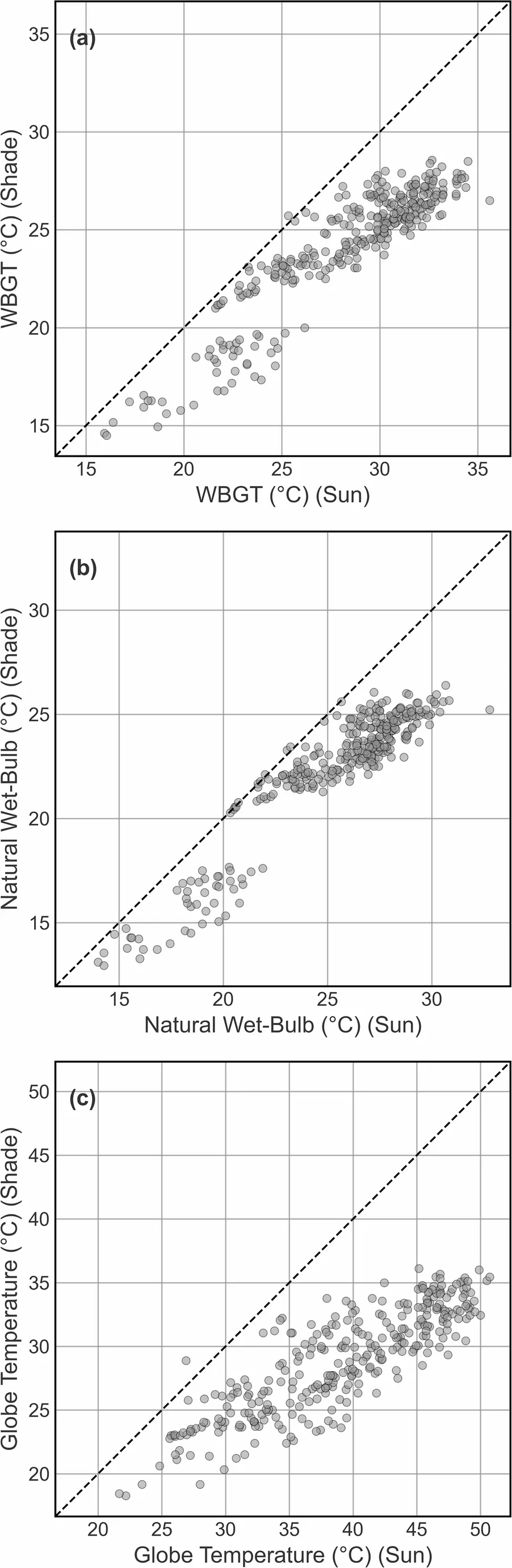
Scatter plots comparing sun-exposed and shaded conditions for: (**a**) wet bulb globe temperature (WBGT), (**b**) natural wet bulb temperature (NWB), and (**c**) globe temperature (GT). Dashed lines indicate the 1:1 reference line

Paired WBGT measurements were compared across the study period to evaluate differences in thermal conditions between sun and shade. As shown in Fig. 2, WBGT values measured in the sun were almost uniformly equal to or greater than those measured in the shade. The mean sun–shade difference in WBGT was 4.13°C (95% CI [3.80–4.45°C]). Examination of the WBGT component variables indicates that both NWB and GT were greater in the sun than in the shade, with particularly large differences observed for GT (Fig. 2b and c). The mean sun–shade difference for NWB was 2.91°C (95% CI [2.66–3.16°C]), while the mean difference for GT was 10.26°C (95% CI [9.54–10.98°C]). Both differences were statistically significant.

**Fig. 3:**
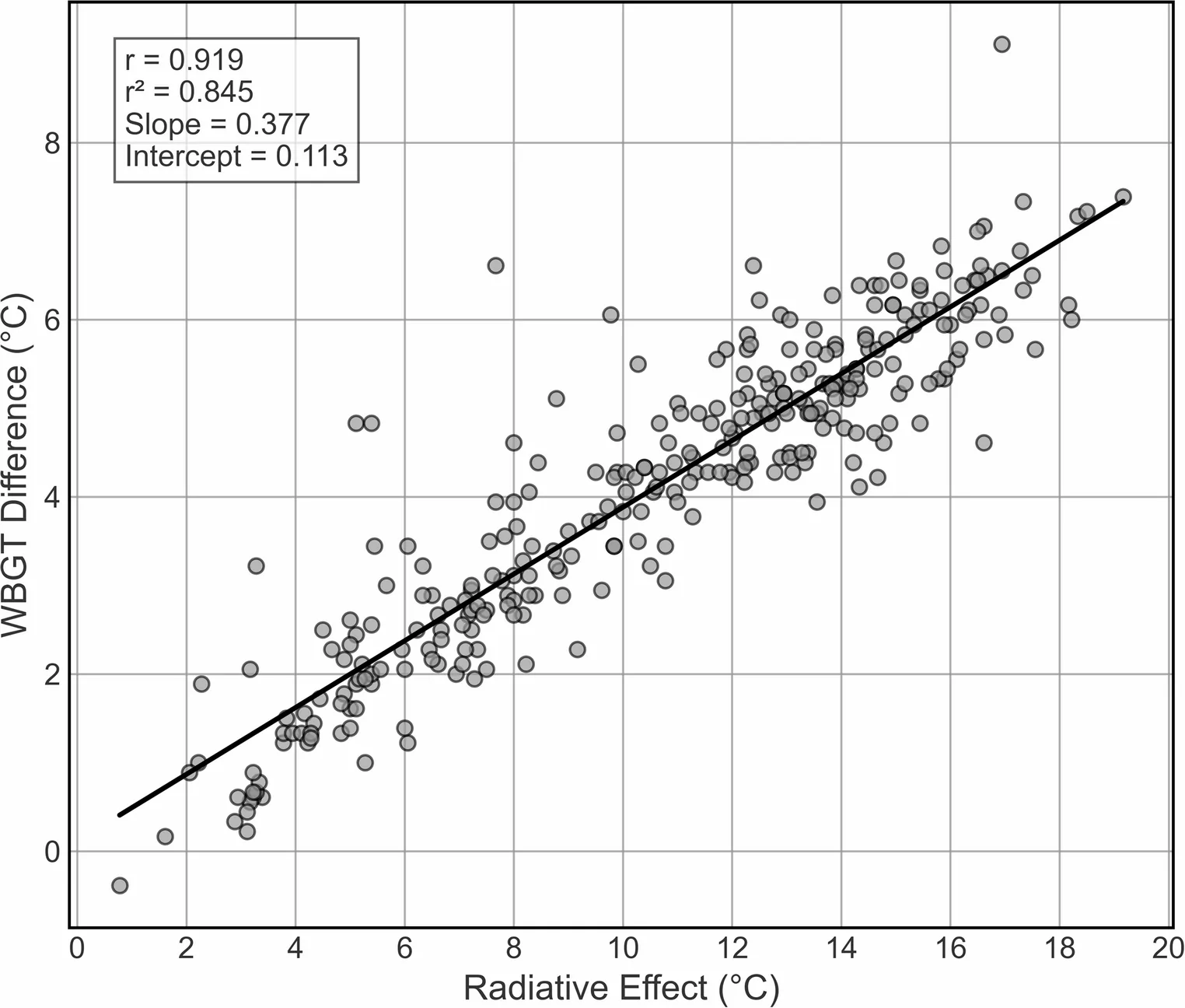
Scatter plot showing the relationship between the radiative effect (globe temperature – dry bulb temperature) in sun-exposed conditions and the corresponding WBGT difference between sun and shaded locations. The solid black line indicates the linear regression line

The radiative effect was strongly associated with the difference between sun and shade WBGT (*r* = 0.92), explaining approximately 85% of the variability in WBGT difference (Fig. 3). The relationship between WBGT difference and radiative effect is described by the regression equation:

$$\:WBGT\hspace{0.17em}=\hspace{0.17em}0.377\:\times\:\:radiative\_effect\hspace{0.17em}+\hspace{0.17em}0.113,$$

where ΔWBGT is the difference between WBGT in the sun and shade (^o^C) and the radiative effect is the difference between GT and DB (^o^C) in the sun. This indicates that every 1°C increase in the radiative effect corresponds to a 0.38°C increase in WBGT. Thus, the average radiative effect over the study period (10.6°C) corresponds to roughly a 4°C increase in WBGT in the sun compared with the shade.

### Microclimatic differences between sites

**Fig. 4:**
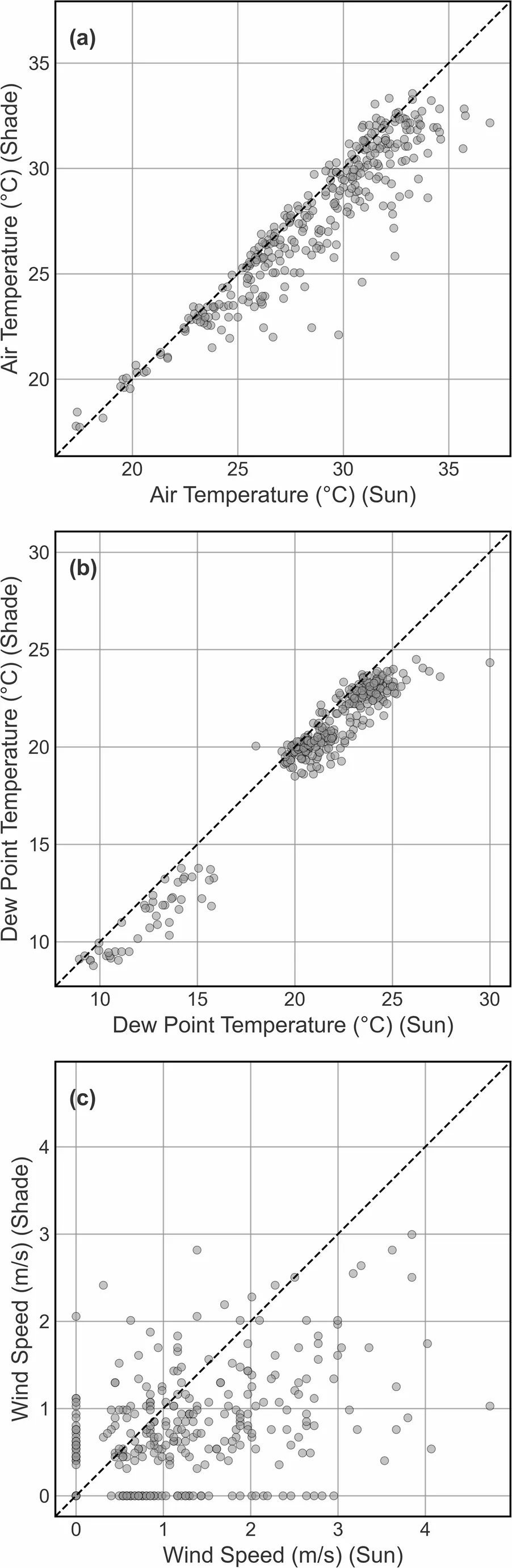
Scatter plots comparing environmental conditions in sun and shade: (**a**) air temperature, (**b**) dewpoint temperature, and (**c**) wind speed. Dashed lines indicate the 1:1 reference line

We compared meteorological differences in air temperature (DB), dewpoint temperature, and wind speed between the sun and shade sites, as these variables influence NWB, GT, and WBGT. As shown in Fig. 4, all three variables were generally higher in the sun than in the shade. The mean sun–shade difference for DB was 1.02°C (95% CI [0.81–1.24°C]), for dewpoint temperature was 0.99°C (95% CI [0.86–1.12°C]), and wind speed was 0.51 m s⁻¹ (95% CI [0.38–0.64 m s⁻¹]).

Because radiative exposure is an important driver of sun–shade thermal differences, we also quantified site differences using the radiant effect term. Radiative effects in the shade were low, averaging 1.41 ± 0.81°C, compared with much higher values in the sun (10.63 ± 4.24°C). The mean sun–shade difference in radiant effect between sun exposed and shaded sites was 9.23°C (95% CI [8.60–9.87°C]). All meteorological variables were statistically significantly greater in the sun than in the shade.

### Mixed model

A linear mixed-effects model indicated that 73% of the variance in the sun–shade WBGT difference (ΔWBGT) was attributable to day-to-day variability (ICC = 0.73). Evaluated at the mean observed meteorological conditions (wind speed = 1.26 m s⁻¹, dewpoint temperature = 20.93°C, and air temperature = 28.49°C), the model-estimated sun-shade WBGT difference was 4.09°C (*p* < 0.001). Wind speed, dewpoint temperature, and air temperature were all significant predictors of ΔWBGT. Specifically, ΔWBGT increased by 0.40°C for each 1 m s⁻¹ increase in wind speed (*p* < 0.001), by 0.48°C for each 1°C increase in dewpoint temperature (*p* < 0.001), and by 0.15°C for each 1°C increase in air temperature (*p* < 0.001).

### Case studies

While we observe pronounced average differences between sun and shade sites, sky cover influences both the temporal variability and the magnitude of WBGT differences. We compared the sun-shade WBGT differences on days with highest (June 28) and lowest (August 9) average radiative effects in the sun. June 28 had the largest average daily radiative effect of 12.91 ± 2.92°C, while August 9 had a lower radiative effect, averaging 6.78 ± 3.26°C, due to greater sky cover.

**Fig. 5:**
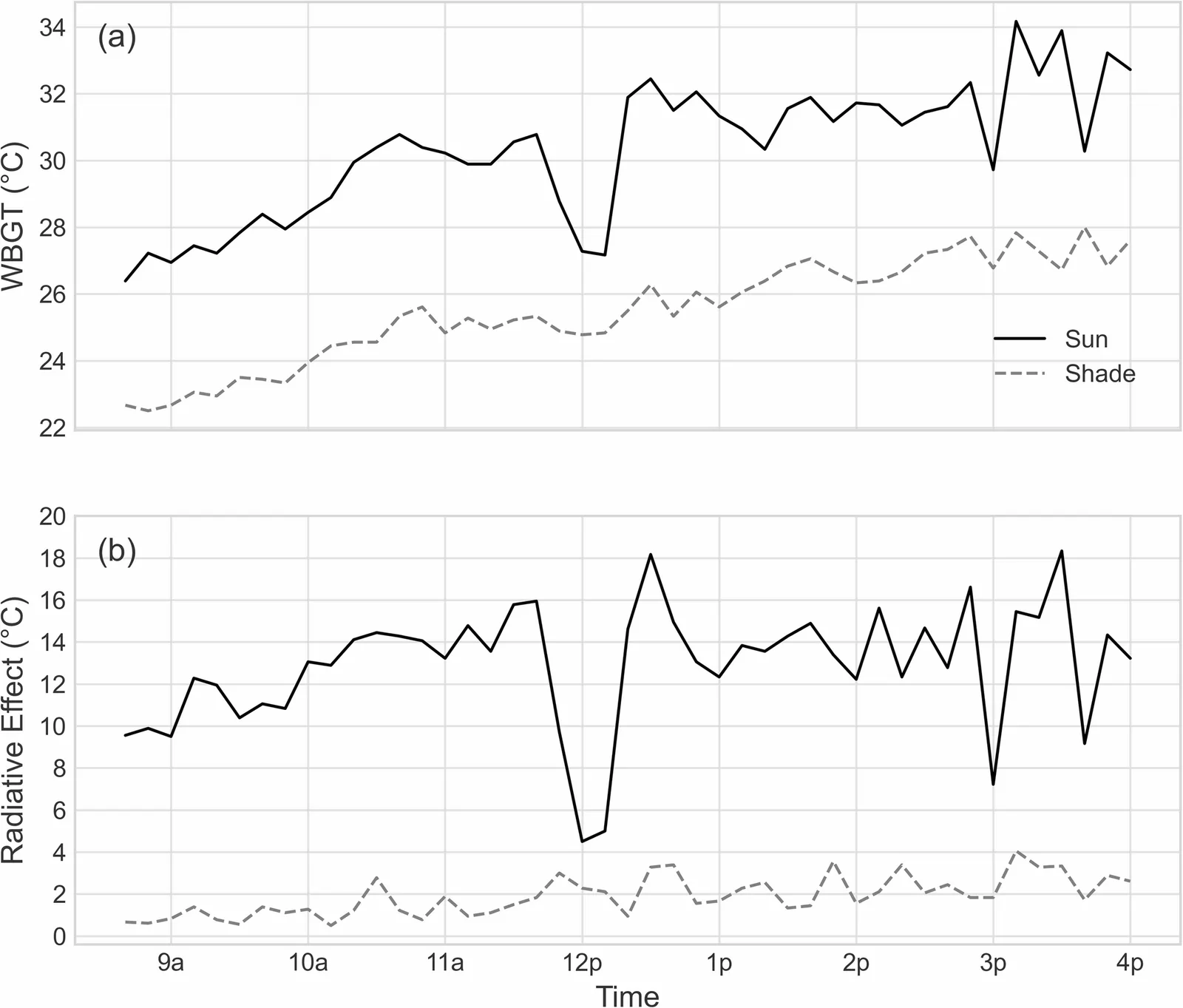
Case study from 28 June 2025: (**a**) wet bulb globe temperature (WBGT) in sun-exposed and shaded locations, and (**b**) radiative effect (globe temperature – dry bulb temperature) in sun and shade

**Fig. 6:**
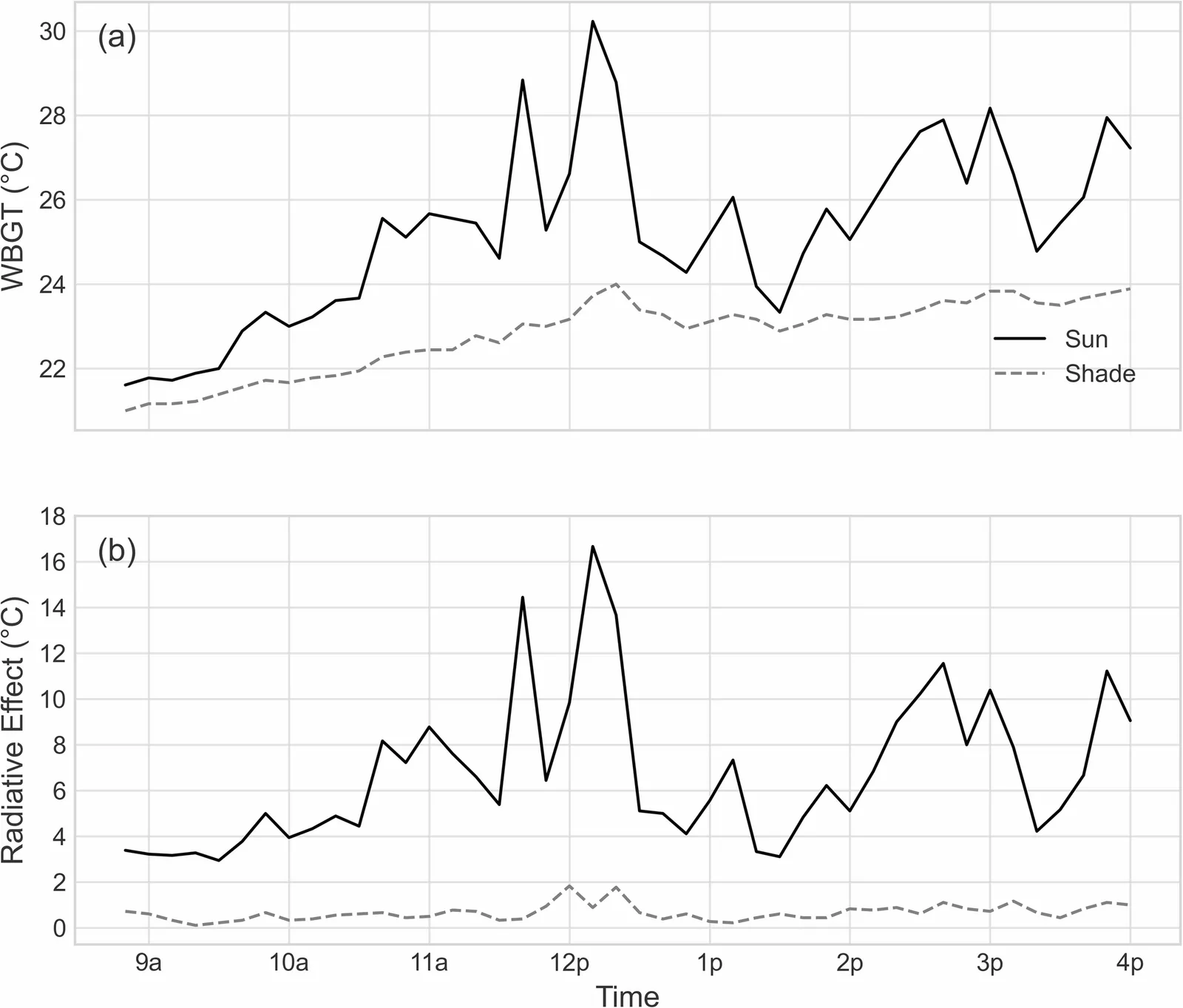
Case study from 9 August 2025: (**a**) wet bulb globe temperature (WBGT) in sun-exposed and shaded locations, and (**b**) radiative effect (globe temperature − air temperature) in sun and shade **Supplement**

The WBGT values for sun and shade over time, along with the radiative effect, are presented in Figs. 5 and 6. On the high-radiation day, the average WBGT difference was 4.82 ± 1.05°C. Sky cover, however, had a substantial influence on this difference (Fig. 5a). For example, on 28 June, which was mostly clear, passing clouds around noon reduced the radiative effect from 15.9°C to 4.5°C, lowering the WBGT difference to 2.5°C (Fig. 5b). This reduction was sufficient to temporarily lower sun-exposed WBGT from above 30°C, where activity modification is recommended under GHSA guidelines, to below 28°C, where no modification is required, before increasing again as skies cleared. On the low-radiative-effect day, the WBGT difference averaged approximately half that of the high-radiation day (2.41 ± 1.42°C) due to greater cloud cover (Fig. 6a). Nonetheless, around noon, brief periods of clearer skies raised the radiative effect to 16.67°C, resulting in a peak WBGT difference of 6.50°C (Fig. 6b). This temporary increase was sufficient to raise sun-exposed WBGT above 28°C, briefly shifting conditions into a category requiring activity modification. Together, these case studies show how short-term changes in solar radiation can alter WBGT-based activity modification recommendations in sun-exposed environments, while shaded conditions remain comparatively stable. We next evaluate the overall influence of shade on GHSA activity modification categories across the full study period.

### Comparison of activity modification category

The WBGT values in the sun and shade were classified into five GHSA categories, ranging from category 1 (no activity modification) to category 5 (cancellation of activities) (Table 2; see Table S1 in Supporting Information). Across the study period, the vast majority of shade conditions (96%) fell into category 1, indicating no need for activity modification, and none of the observations in the shade fell into categories 3–5. In contrast, only 36% of sun-exposed conditions were in category 1, meaning that 64% required some form of activity modification (categories 2–5), with approximately 15% of sun-exposed observations falling into category 4 (strict activity modification) or category 5 (cancellation). There was a significant difference in WBGT-based activity modification between sun and shade conditions, χ²(4, *N* = 309) = 255.49, *p* < 0.001.

**Table 2:** Frequency of activity modification categories based on corresponding WBGT

GHSA Category^1^	Sun Count (%)	Shade Count (%)
1	111 (35.9)	298 (96.4)
2	86 (27.8)	11 (3.6)
3	65 (21.0)	0 (0.0)
4	35 (11.3)	0 (0.0)
5	12 (3.9)	0 (0.0)

^1^See Supplemental Table 1 for details on the GHSA categories

## Discussion

This study provides empirical evidence of the impact of a permanent shade pavilion on WBGT in a recreational sport setting. Previous studies have consistently shown that shading reduces daytime thermal stress, resulting in lower mean radiant temperature and consequently reducing values of thermal comfort indices such as the Physiological Equivalent Temperature (PET) and Universal Thermal Climate Index (UTCI) (e.g., Middel et al. 2021; Lam et al. 2023; Aleksandrowicz and Pearlmutter 2023; Ouyang et al. 2024; Jo and Park 2025). Our findings align with this body of work while extending it by explicitly quantifying shade effects using WBGT, the primary heat stress metric used to guide activity modification in sport and recreation.

Consistent with prior research, air temperature under the pavilion was modestly lower than in full sun, with an average reduction of approximately 1°C. This magnitude corresponds with previous studies reporting air temperature reductions of less than 2°C in shaded environments (Middel et al. 2021; Ouyang et al. 2024), reinforcing that the primary cooling benefit of shade during daytime conditions is not driven by large changes in air temperature alone. Rather, daytime sun–shade differences in WBGT are primarily drive by reductions in radiative heating, as shading directly reduces incoming solar radiation while having a limited effect on air temperature.

As such, evaluating how shading modifies radiative heating is important for understanding its influence on WBGT. Previous studies have commonly quantified radiative effects using mean radiant temperature (MRT), which can be reduced by approximately 15–40°C depending on time of day and shading configuration (e.g., Middel et al. 2021; Aleksandrowicz and Pearlmutter 2023; Ouyang et al. 2024). In contrast, this study quantified radiative heating using the radiative effect term (GT–DB), which incorporates the same components used to calculate WBGT. We observed an average 9°C reduction in radiative heating under the shade pavilion, and this reduction was directly related to WBGT, with a 1°C increase in radiative effect corresponding to a 0.38°C increase in WBGT.

Sensor placement likely influenced the magnitude of observed radiative reductions. Sensors were positioned near the edge of the pavilion and, although not exposed to direct sunlight, likely experienced greater diffuse radiation than would occur toward the center of the structure. In larger, football-sized shade pavilions or under more centrally located shaded conditions, diffuse radiation exposure would be further reduced. Consequently, the radiative and WBGT differences observed here are likely conservative, and the true cooling benefit of full-coverage shade may be larger than reported.

In terms of WBGT, shading produced substantial reductions, with a mean adjusted decrease of approximately 4°C after accounting for the microclimatic differences between sensors. The magnitude of the WBGT reduction varied with sky conditions, with larger sun–shade differences observed under clear-sky conditions when solar radiation was strongest. From our case study, average WBGT reductions were 4.8°C on a sunny day and 2.4°C on a cloudier day, consistent with prior findings that cooling benefits of shading are greater under high-radiation conditions. Watanabe and Ishii (2017), for example, reported WBGT reductions from parasol shade of 0.9–1.8°C under sunny conditions and 0.5–1.0°C under cloudy conditions. The larger reductions observed in our study likely reflect the full-coverage design of the permanent shade pavilion, as more extensive shade structures are more effective at reducing radiative heat loads than smaller or lightweight shading devices (Middel et al. 2021).

Two considerations are important when interpreting the WBGT reductions beneath the pavilion. First, the shaded and sun-exposed measurement locations differed not only in solar exposure but also in underlying surface type. The pavilion was situated over concrete, whereas the sun-exposed comparison site was located over grass. Consequently, the 4.09°C WBGT reduction reflects the combined effect of shading and the site-specific surface configuration, and the independent contribution of shade cannot be isolated. Future studies using matched surface types under both shaded and sun-exposed conditions are needed to separate the effects of shading and substrate on WBGT. Second, the model estimate depends on the meteorological covariates included in the analysis. Air temperature, dewpoint temperature, and wind speed were measured outside the pavilion at the sun-exposed grass site, representative of outdoor sports fields. The 4.09°C estimate is therefore best characterized as a sun–shade contrast conditional on mean observed local meteorological conditions at this site, rather than an isolated causal effect of shade alone.

These WBGT reductions translated into meaningful differences in activity-modification categorizations. Nearly all shaded observations (96%) fell into GHSA categories requiring no activity modification, compared with only 36% of observations in full sun. When translated to a standardized 9 a.m. to 4 p.m. recreation period, these findings indicate a substantial extension of time without required activity modification. On average, conditions under the pavilion met the criterion for no activity modification for 6.7 h, compared with only 2.5 h in adjacent sun-exposed areas, representing an additional 4.2 h of unrestricted activity time. Additionally, 15% of sun-exposed observations reached the most restrictive activity modification categories, whereas no such conditions occurred under the pavilion. Thus, WBGT reductions on the order of 3–5°C were sufficient to shift a large proportion of conditions below key activity modification thresholds and expand opportunities for outdoor programming. Although these findings are based on observations at a single structure over a limited observation period, they suggest that permanent shade infrastructure has the potential to produce similarly meaningful shifts in activity-modification categorizations at other sport and recreational facilities. These results correspond with broader public health and climate-adaptation recommendations that identify expanded shade access as an effective strategy for reducing heat exposure in outdoor environments. As schools, municipalities, and recreation agencies continue to invest in shade infrastructure, permanent shade structures may represent a practical adaptation measure that complements approaches such as schedule modifications, activity restrictions, and indoor relocation. Additional studies across different climates, structure designs, and seasons are needed to assess the generalizability of these benefits.

Finally, shade structures may play a complementary role in supporting heat acclimatization by selectively reducing radiative heat load while preserving exposure to elevated ambient air temperatures. Heat acclimatization develops through repeated heat exposure over approximately 7–14 days and decays rapidly in the absence of continued outdoor heat stress (Armstrong et al. 1991; Garrett et al. 2011; Daanen et al. 2018). Unlike fully climate-controlled environments, shaded outdoor settings maintain thermal exposure sufficient to support acclimatization while lowering WBGT and associated heat illness risk. In this context, permanent shade structures may function as a practical risk-management tool that balances heat safety with the maintenance of physiological heat adaptation.

## Limitations

While this study provides empirical data on the performance of a permanent shade pavilion in reducing WBGT under a range of meteorological conditions, several limitations should be acknowledged. First, the findings reflect a humid subtropical climate. Although data collection was restricted to seven study days, this monitoring window successfully captured a broad range of intra-regional meteorological variability, ranging from typical to extreme temperature, humidity, and cloud-cover conditions (as detailed in Sect.  3.1). Therefore, while these results are representative of the diverse conditions within a humid subtropical summer, full generalizability may be limited in climates characterized by substantially different environmental conditions, such as hot, arid environments. Second, the results are specific to this particular pavilion design, including its size, height, material, openness, and orientation. Additional research is needed to evaluate how other pavilion configurations perform under comparable conditions. Third, the pavilion was situated over concrete while the sun-exposed sensor was positioned over grass; the independent contributions of shading and substrate characteristics therefore cannot be fully disentangled (see Discussion). Fourth, sensors were positioned near the edge of the pavilion, which, although not in direct sunlight, likely increased exposure to diffuse radiation. As a result, observed sun–shade differences in WBGT are likely conservative. Finally, this study evaluated a passive shade pavilion without mechanical ventilation. Because increased air movement can enhance evaporative cooling and reduce NWB, future work should quantify the combined benefits of fans and shading for greater heat-stress relief and improved thermal comfort.

## Conclusions

Our study demonstrates that substantial reductions in WBGT can be achieved by relocating athletic activities from direct sun exposure to a permanent shaded pavilion. These cooler conditions have practical benefits for sports and physical activities, allowing more frequent participation in normal activities and reducing the need for activity modifications, including cancellations. The dataset encompassed a wide range of meteorological conditions, suggesting that the benefits of shading are consistent across varied environmental settings. Nevertheless, further research is needed in different climates, with alternative pavilion designs, and over consistent surface types to generalize these findings.

## Supplementary Information

Below is the link to the electronic supplementary material.

Supplementary Material 1

## Data Availability

The datasets generated during and/or analyzed during the current study are available from the corresponding author on reasonable request.
