# Supplementary material for: Quantifying the influence of a permanent shade pavilion structure on WBGT and activity modification
Source: Int J Biometeorol. 2026 Oct 28;70(11):287. doi: 10.1007/s00484-026-03345-8 (PMC13719781; doi:10.1007/s00484-026-03345-8)
Supplement: Supplementary file 1 [file 484_2026_3345_MOESM1_ESM.docx]

**Supplemental Table 1**: Georgia High School Association WBGT Activity Modification Guidelines.

| **WBGT (^o^C)** | **WBGT (^o^F)** | **Guidelines** |
| --- | --- | --- |
| <27.78 | <82.0 | Normal Activities - Provide at least three separate rest breaks each hour with a minimum duration of 3 minutes each during the workout. |
| 27.78-30.50 | 82.0-86.9 | Use discretion for intense or prolonged exercise; watch at-risk players carefully. Provide at least three separate rest breaks each hour with a minimum duration of 4 minutes each. |
| 30.51-32.17 | 87.0-  89.9 | Maximum practice time is 2 hours. For Football: players are restricted to helmet, shoulder pads, and shorts during practice, and all protective equipment must be removed during conditioning activities. If the WBGT rises to this level during practice, players may continue to work during out wearing football pants without changing to shorts. For All Sports: provide at least four separate rest breaks each hour with a minimum duration of 4 minutes each. |
| 32.18-33.33 | 90.0-92.0 | Maximum practice time is 1 hour. For Football: no protective equipment may be worn during practice, and there may be no conditioning activities. For All Sports: there must be 20 minutes of rest breaks distributed throughout the hour of practice. |
| >33.33 | >92.0 | NO OUTDOOR WORKOUTS. Delay practice until a cooler WBGT level is reached. |
